# Cadaveric emergency cricothyrotomy training for non-surgeons using a bronchoscopy-enhanced curriculum

**DOI:** 10.1371/journal.pone.0282403

**Published:** 2023-03-23

**Authors:** Caterina Zagona-Prizio, Michael A. Pascoe, Michaele Francesco Corbisiero, Violette C. Simon, Scott E. Mann, Katherine A. Mayer, James P. Maloney

**Affiliations:** 1 School of Medicine, University of Colorado Anschutz Medical Campus, Aurora, CO, United States of America; 2 Department of Otolaryngology Head and Neck Surgery, University of Colorado, Aurora, CO, United States of America; 3 Department of Surgery, Denver Health Medical Center, Denver, CO, United States of America; 4 Department of Emergency Medicine, University of Colorado Anschutz Medical Campus, Aurora, CO, United States of America; 5 Pulmonary Sciences and Critical Care Medicine, University of Colorado Anschutz Medical Campus, Aurora, CO, United States of America; Sapienza University of Rome: Universita degli Studi di Roma La Sapienza, ITALY

## Abstract

**Background:**

Emergency cricothyrotomy training for non-surgeons is important as rare “cannot intubate or oxygenate events” may occur multiple times in a provider’s career when surgical expertise is not immediately available. However, such training is highly variable and often infrequent, therefore, enhancing these experiences is important.

**Research question:**

Is bronchoscopy-enhanced cricothyrotomy training in cadavers feasible, and what are the potential benefits provided by this innovation for trainees?

**Methods:**

This study was performed during implementation of a new program to train non-surgeon providers on cadaveric donors on our campus. Standard training with an instructional video and live coaching was enhanced by bronchoscopic visualization of the trachea allowing participants to review their technique after performing scalpel and Seldinger-technique procedures, and to review their colleagues’ technique on live video. Feasibility was measured through assessing helpfulness for trainees, cost, setup time, quality of images, and operator needs. Footage from the bronchoscopy recordings was analyzed to assess puncture-to-tube time, safety errors, and evidence for a training effect within groups. Participants submitted pre- and post-session surveys assessing their levels of experience and gauging their confidence and anxiety with cricothyrotomies.

**Results:**

The training program met feasibility criteria for low costs (<200 USD/donor), setup time (<30 minutes/donor), and operator needs (1/donor). Furthermore, all participants rated the cadaveric session as helpful. Participants demonstrated efficient technique, with a median puncture-to-tube time of 48.5 seconds. Bronchoscopy recordings from 24 analyzed videos revealed eight instances of sharp instruments puncturing the posterior tracheal wall (33% rate), and two instances of improper tube placement (8% rate). Sharp instruments reached potentially dangerous insertion depths beyond the midpoint of the anterior-posterior diameter of the trachea in 58.3% of videos. Bronchoscopic enhancement was rated as quite or extremely helpful for visualizing the trachea (83.3%) and to assess depth of instrumentation (91.7%). There was a significant average increase in confidence (64.4%, *P*<0.001) and average decrease in performance anxiety (-11.6%, *P* = 0.0328) after the session. A training effect was seem wherein the last trainee in each group had no posterior tracheal wall injuries.

**Interpretation:**

Supplementing cadaveric emergent cricothyrotomy training programs with tracheal bronchoscopy is feasible, helpful to trainees, and meets prior documented times for efficient technique. Furthermore, it was successful in detecting technical errors that would have been missed in a standard training program. Bronchoscopic enhancement is a valuable addition to cricothyrotomy cadaveric training programs and may help avoid real-life complications.

## Introduction

Difficult airway management may require rescue procedures such as a cricothyrotomy to be performed by non-surgeon providers. Current guidelines recommend cricothyrotomy as a last resort procedure in situations when endotracheal intubation is not possible and the patient cannot be oxygenated [1, 2]. Cricothyrotomies establish an airway by puncturing the cricothyroid membrane, and can be necessary in patients with facial trauma, angioedema, laryngeal tumors, morbid obesity, trismus, burns, and other causes of upper airway obstruction [3]. Cricothyrotomies are frequently preferred to tracheostomies by non-surgeons due to their rapidity and simplicity, but inexperience and inaccurate execution results in variable success rates and increased complications [4–6]. Common complications include posterior tracheal wall injury and improper tube placement [7, 8]. A traumatic complication on an otherwise “successful” procedure might decrease the confidence and willingness of non-surgeon providers to perform this procedure in the future.

Despite the importance of proficiency in performing a cricothyrotomy during life-threatening situations, there are several challenges that residency and fellowship programs face in providing training. Notably, the procedure is rarely performed, limiting training opportunities for non-surgeons to gain experience with live patients; additionally, there are ethical considerations to training on recently deceased patients and live animals [9–11]. Cadaveric training has been shown to be superior to simulation training due to landmark and tissue fidelity, yet few local or regional training programs for non-surgeons have access a limited supply of expensive to cadaveric donors [9, 12–14]. Finally, the skills required to perform a cricothyrotomy decline over time, necessitating regular re-training to maintain comfort and proficiency [15]. These hurdles result in inconsistent training for non-surgeons across residency, fellowship, and hospital programs.

Given the limited access to cadavers, it is critical to provide the most robust curricula possible when such cadaveric training is available. As a result, we chose to develop a first ever cadaveric cricothyrotomy training program on our campus and augment it with live bronchoscopic visualization of the trachea during procedures. The benefits of intraprocedural bronchoscopy during percutaneous tracheostomy, particularly for surgeons in training, have been well-recognized for over a decade. Posterior tracheal wall injuries and esophageal perforations are well-recognized complications of typical (Seldinger) percutaneous tracheostomy techniques [16]. Intra-procedure bronchoscopy is recommended as part of a bundle to reduce such complications [17]. We realized this technology might aid emergency cricothyrotomy training in a similar manner. Moreover, the recent appearance of lost-cost disposable bronchoscopes offered the opportunity to accomplish such training without the prohibitively high acquisition costs for obtaining and maintaining multiple non-disposable bronchoscopes. No previous study to our knowledge has provided bronchoscopic feedback during emergency cricothyrotomy training. We hypothesized this training feature would be a feasible addition while meeting accepted times for achieving an airway published in the literature. Furthermore, we hypothesized it would provide a useful means to detect and prevent intra-tracheal complications, and be valued by trainees.

## Methods

### Study design

We implemented a new program to train non-surgeon fellows and attendings on cadaveric donors assigned to the Physical Therapy and Modern Human Anatomy programs at the University of Colorado School of Medicine. Donors were sourced from the Colorado State Anatomical Board (https://medschool.cuanschutz.edu/state-anatomical-board. These formaldehyde-embalmed donor cadavers were selected as their necks were not yet dissected during this graduate student educational program and were provided *pro bono* for use in our training program. Participants were invited to one of three training sessions held between August 2020 and June 2021, resulting in a total of 24 participants. Of the 24 participants, 13 were critical care attending physicians, nine were pulmonary and critical care fellows, and two were anesthesiology critical care fellows. The training was initially conceived to have larger training groups of six, but due to the limitations of the COVID-19 pandemic, larger participant groups were separated into smaller cohorts of three to maintain physical distancing requirements. These experimental procedures were reviewed and qualified for exemption from federal regulations by the Colorado Multiple Institutional Review Board (Protocol #20–2303; including cadaver and trainee aspects) and were approved by the Colorado State Anatomic Board.

The training program’s objectives were identified by a team of medical educators from fields of critical care medicine, emergency medicine, otolaryngology, and anatomy. The primary objective was to design a cadaveric cricothyrotomy training program that would meet or exceed prior documented times for efficient technique while assessing the feasibility of enhancing training utilizing live bronchoscopic imaging. Feasibility was defined as meeting all of the following parameters: the majority of trainees rating bronchoscopy as helpful to their learning in a post-training survey, cost maintained at under 200 USD/donor, set-up time per donor under 30 minutes, and one operator (beyond the instructor) being sufficient to maintain scope position and high-quality bronchoscopy imaging for both live feedback and recordings. Efficient technique was defined as performing the surgical (scalpel) technique and the kit (Seldinger) technique meeting puncture-to-tube times (PTt) at or below times reported by other published training programs, and placement of the endotracheal tube in the tracheal lumen (success rate). We selected 95 seconds for both techniques as these times were similar to existing reports [18–27].

The secondary objectives assessed were intra-tracheal complication detection, quality of the training program, and a preliminary assessment of training effect. Complications were measured by analyzing the captured bronchoscopy videos for potentially dangerous sharp instrument insertions, number of punctures to the posterior tracheal wall, and any other unanticipated complications such as extraluminal tube or bougie placement. We defined potentially dangerous insertion depths as insertion of sharp instruments beyond the midpoint of the anterior-posterior diameter of the trachea. This criterion was selected since substantial anterior movement of the posterior wall of the trachea could be expected with the respiratory efforts of alive, non-intubated patients. We felt that the midpoint was a conservative guardrail for safety for trainees. Punctures or damage to the posterior tracheal wall visualized on the monitors were not confirmed by dissection and pathologic examination as most of the donors were used for full head and neck dissections by anatomy students (with divergent training needs) in subsequent days.

Quality of the program was evaluated through the Kirkpatrick model for evaluating training programs [28]. For this pilot study we chose to assess levels one (reaction) and two (learning) through a pre- and post-survey measuring helpfulness to participants (reaction) and whether confidence improved and anxiety lessened (learning). Training effect was measured by assessing posterior tracheal wall injury rates between trainee sequence within group of 3 participants rotating together (trio), hypothesizing that the third in the trio would benefit from watching bronchoscopic footage for the first two trainees and have lower injury rates.

Session

Ten disposable bronchoscopes (slim 3.8, regular 5.0, and large 5.6 sizes; Ambu Inc, Columbus, MD, USA) were purchased for the sessions at a cost of 160 USD each through veterinary surplus companies. The remaining scopes were provided *pro bono* by Ambu. Prior to each session, one bronchoscope per donor were placed orally and advanced to just inferior to the vocal cords in order to visualize the superior trachea. Three faculty members (JM, KM, SM) placed the scopes for each session. Due to upper airway debris, portable suction and water lavage was typically required to clear upper airways and place bronchoscopes. Bronchoscopes were attached to proprietary AMBU portable monitors on sturdy rolling IV poles which were both borrowed from our hospital. These monitors were moved between donors to follow the trainees as they moved through their sessions. Volunteer medical students (CZP, VS, MFC) and a faculty physician (JPM) moved between the donors to assist in maintaining optimal bronchoscope positioning for each trainee and to record videos.

The video screen was not visible to the trainee performing the procedure, but was available for real-time monitoring by the other two trainees in the training trio. The two trainees clustered around the monitor screen and helped communicate to the trainee whenever they hit the posterior tracheal wall with a sharp instrument, and thus were influenced on safe sharp instrument technique before performing their own procedure. In the original design group size would have been six, but after onset of the COVID-19 pandemic (before training sessions began) our campus restricted group sizes to three trainees and two educational staff, so only 2 of 3 trainees (instead of the 5 of 6 originally intended) in the trio were able to benefit from seeing the live bronchoscopic video before doing their own procedure.

**Fig 1:**
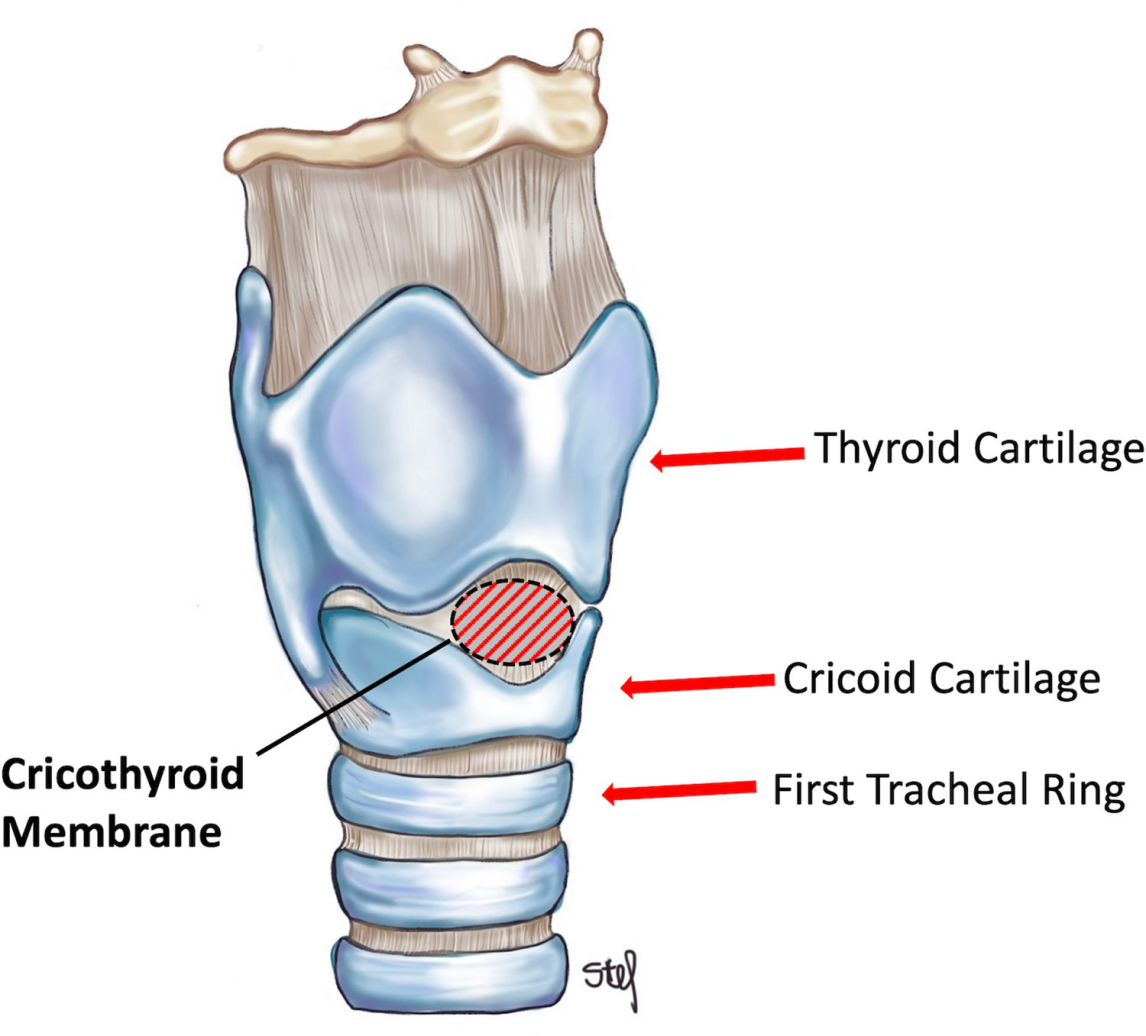
Overview of laryngeal and upper tracheal anatomy.

Participants were instructed to review an instructional video before the session. The video contained illustrations of tracheal anatomy (**Fig 1**) and information on basic scalpel and Seldinger techniques, as well as strategies for performing a cricothyrotomy on patients with difficult neck anatomy. The scalpel technique used a disposable scalpel, bougie (15 French x 70 cm, Medline Industries, Inc.; Northfield, IL, USA) and 6.0 endotracheal tube (Medline) and was performed in all cases with an attending surgeon as the instructor (SM) across from the trainee. The Seldinger technique used a kit (Melker 4.0 cuffed, Cook Medical; Bloomington, IN, USA) and was performed in all cases with an attending emergency medicine attending as the instructor (KM) across from the trainee. The video was produced with funds from a local educational grant and was published to YouTube and Zenodo websites as an open-access resource for other training programs [29, 30].

Participants began the session by completing a pre-session survey. Trainees were able to opt out of the surveys and research study without penalty (none did so). They then reviewed cricothyrotomy basic techniques with their airway experts and were instructed to avoid deep sharp instrument depths with scalpel blades or after aspirating air (Seldinger technique). Each trainee performed the scalpel technique first on their assigned donor which was assisted by the airway expert who passed them the bougie and endotracheal tube. They were provided with immediate feedback on unsafe instrument depth observed on live bronchoscopy (hitting the posterior tracheal wall) by the staff (and the other two trainees) monitoring live video in order to maximize the potential safety benefits of this training program for cricothyrotomies performed on future living patients. Live images were not shown to trainees during their attempts as such information would not replicate the clinical scenario. After achieving the emergency airway, each trainee reviewed their attempt on the video recordings captured on the bronchoscope monitor, after all three trainees had completed procedures. Procedural success was defined as endoscopic confirmation of an airway in the trachea. Bronchoscopic video data files are available for review using website links posted in the Supplemental Data section at the end of this manuscript.

Next, the participants performed an unassisted Seldinger technique on another donor, posterior tracheal wall violations were similarly called out, and again trainees reviewed the procedure on the monitor after the group was done. Thus, 48 donors were used between all trainees. Subsequently, trainees were instructed to repeat the steps of the procedure where the airway experts felt that they were not proficient (e.g. scalpel insertion depth). These subsequent procedures were not recorded as “attempts” nor were they evaluated as data in the study as they were performed through existing incisions. After the session the participants completed a post-session survey.

### Data collection

#### Video

**Fig 2:**
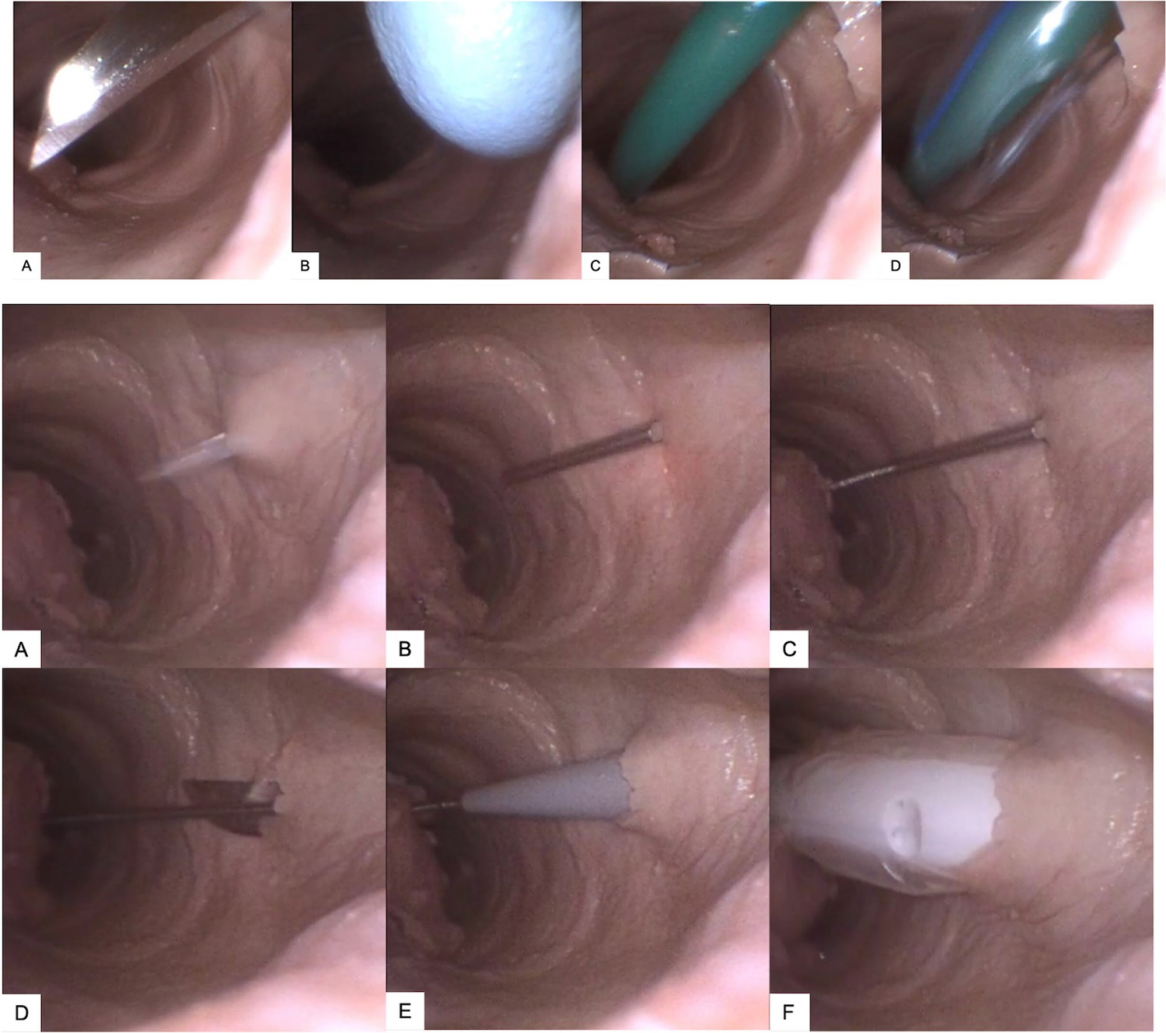
**A**. **Representative bronchoscopic images of scalpel technique**. (A) Scalpel insertion into tracheal lumen; (B) Blunt plastic end of scalpel seen in tracheal lumen, enlarging scalpel incision before insertion of bougie; (C) Bougie insertion; (D) Insertion of 6.0 endotracheal tube over bougie. **B**. **Representative bronchoscopic images of Seldinger technique.** (A) Needle insertion; (B) Needle appropriately directed inferiorly before wire insertion; (C) Wire advancing through trocar needle; (D) Scalpel enlarging trocar needle incision before dilator insertion; (E) Introducer tip placed over wire; (F) Tracheostomy tube and balloon entering tracheal lumen.

**Fig 3:**
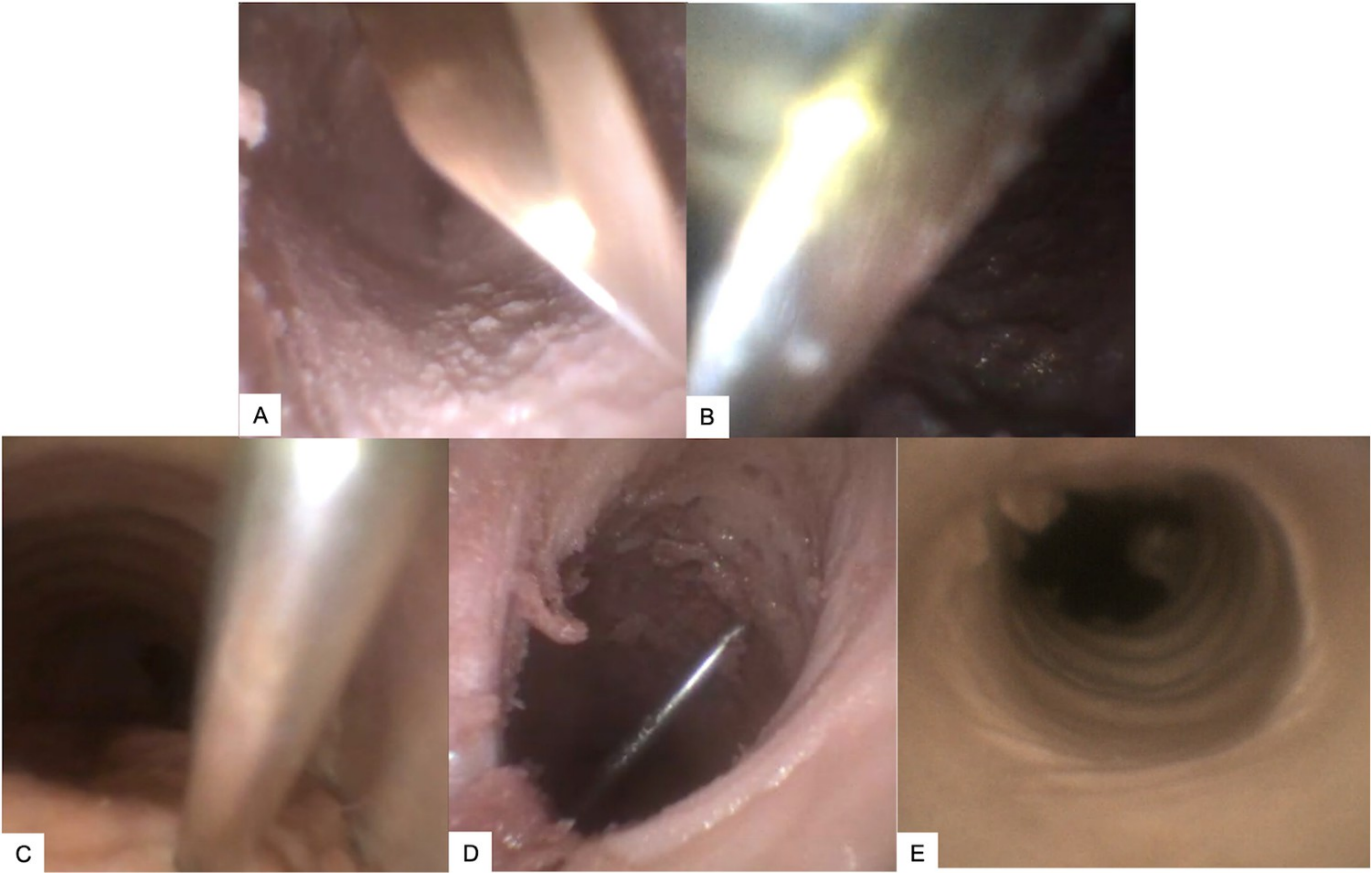
Bronchoscopic capture of complications with depiction of the midpoint of the anterior-posterior depth of the trachea. (A) Scalpel puncture of posterior tracheal wall at 4 o’clock position; (B) Scalpel puncture of posterior tracheal wall at 7 o’clock position; (C) Needle puncture of posterior tracheal wall at 6 o’clock position; (D) Needle puncture of posterior tracheal wall at 7 o’clock position; (E) Absence of bougie catheter in trachea after being placed lateral to trachea.

Twenty-four high-quality bronchoscopy recordings from the sessions were saved for analysis. The most common reasons for not having a high-quality recording throughout the procedures were anterior collapse of the trachea during instrument and tube insertion, and debris movement in the trachea. Two reviewers (JPM, MFC) examined the 24 videos to determine the number of punctures to the posterior tracheal wall, and for any other unanticipated complications such as lateral wall punctures or extraluminal tube bougie placement. Captured stills from these endoscopic views demonstrate proper technique (**Fig 2A and 2B**) and complications (**Fig 3**). One trio of trainees videos were not timestamped and therefore excluded from the complication rate trio analysis of training effect. PTt was calculated from the time of initial instrument appearance (trocar needles or scalpels) in the anterior tracheal wall until endotracheal tube placement with a visible cuff from nineteen of these videos with optimal views for the exact duration of the procedure. A third reviewer (CZP) analyzed any inconsistencies noted between the first two for the final scoring. The PTts and injury rates captured from this study were compared with times from 10 other studies that served as a control group for procedure time [18–27].

#### Survey

The pre-session survey assessed the participants’ level of experience with performing cricothyrotomies from prior simulation environments and on living patients during residency or fellowship training. It also assessed participants’ confidence and anxiety with performing the procedure. Survey questions were developed specifically for this program as we are unaware of any validated instruments assessing anxiety or confidence for post-graduate procedural (non-surgical) training. Levels of confidence and anxiety were measured on a 4-point Likert scale. The scale for anxiety included: not anxious at all (1), slightly anxious (2), moderately anxious (3), extremely anxious (4). The scale for confidence was similar: not at all confident (1), slightly confident (2), moderately confident (3), extremely confident (4). Immediately after the session trainees completed a post-survey that measured the utility of the training video and of the bronchoscopic feedback, both for the recorded first attempt and for the live feedback during subsequent attempts. A similar 4-point Likert scale was used: not helpful at all (1), somewhat helpful (2), quite helpful (3), extremely helpful (4). The survey also re-assessed their confidence and anxiety for performing cricothyrotomies in the context of bronchoscopy-enhanced training.

### Data analysis

Data were imported into a spreadsheet (Microsoft Excel, version 16.55, Microsoft Corp., Redmond, WA) for organization and cleaning. An alpha level of *P*<0.05 was defined to identify significant differences and statistical analyses were performed using STATA 15.1 (StataCorp LP, College Station, TX). Descriptive statistics were calculated for complications and PTt. Chi-squared analysis was performed to evaluate training effect within trios. A two-sample, unpaired t-test was used to analyze the average PTt for the scalpel and Seldinger techniques. Wilcoxon signed-rank tests were run on pre- and post-session survey data assessing confidence and anxiety with performing an emergency cricothyrotomy. Univariate regression analysis was applied to assess the effects of inexperience on anxiety and comfort with the procedure.

## Results

Costs for the training program were kept low through using disposable bronchoscopes (160 USD/scope). Moreover, we found that each scope was re-usable on average twice before maneuverability and image quality degraded. In comparison, using non-disposable bronchoscopes would not have been feasible given their cost at thousands of USD each. The borrowed proprietary monitors additionally provided high quality live images and recorded well for playback and data downloads, other than occasional loss of sequence stamps within training trios. The time required to place scopes proved to be reasonable, generally taking 5–15 min to prepare the airway and place the scope when using suction and tap water lavage (without suction, this time could double). Five donors had substantial upper airway obstruction from debris that could not be cleared and were not used for training. In a session with twelve donors, three airway experts could place twelve bronchoscopes during one hour. Placing the scopes before the session was essential to keep trainee in-room time efficient which would have not been feasible with non-disposable scopes due to the cost of acquiring such a large number of bronchoscopes. One operator per individual attempt was able to acquire video recordings and maintain the bronchoscopes in optimal position during the sessions (with occasional assistance from an airway expert) as the pressure from palpation, sharp instruments, and tube placement often affected optimal bronchoscope position.

PTts ranged from 22.5 to 135 seconds; the mean for the videos was 61.9±34.2 seconds (*M* ± *SD*), and the median was 48.5 seconds. Outliers with longer durations were included in the mean and median calculations and were associated with multiple instrument insertions or complications in tube placement reflecting prolonged teaching time. Compared to the scalpel technique (*M* = 35.5, *SD* = 11.0), the Seldinger technique (*M* = 91.3, *SD* = 25.5) had a significantly longer mean PTt, *t*(17) = 6.329, *P* = 0.0001 (**Table 1**). Only first-attempt techniques were evaluated for these data (only one trainee did not obtain an airway). These PTts compared favorably with other procedure times reported in the literature (**Table 2**).

**Table 1:** Puncture-to-Tube time stratified by cricothyrotomy technique.

	Puncture-to-Tube time (PTt)^a^		
Assessed Videos	Mean ± SD (seconds)	*P*-value	Median (seconds)
All videos^b^	61.9±34.2		48.5
Technique			
Scalpel	35.5±11.0		35.0
Seldinger	91.3±25.5		90.5
Difference in means^c^	55.8	0.0001	

SD = standard deviation; PTt = puncture-to-tube time

^a^ PTt defined as the time of initial instrument appearance (trocar needles or scalpels) in the anterior tracheal wall until endotracheal tube placement with a visible cuff.

^b^ All videos denotes all of the videos assessed with a full view of the trachea for the duration of the procedure, regardless of technique

^c^ Difference in means between the scalpel and Seldinger techniques

**Table 2:** Published cricothyrotomy training programs using human cadavers: Characteristics and data.

				Surgical Technique			Seldinger Technique				
First Author	Year	#	Educational Level (N)	PTt (secs)	Success Rate	Posterior Tracheal Injury	PTt (secs)	Success Rate	Posterior Tracheal Injury	Live Endoscopy Feedback	Injury Exam (dissection)
Holmes	1998	32	residents (32)	43	88%	9%	134	94%	3%	No	No
			[emergency]								
Chan	1999	30	residents (13)	73	87%	0%	75	93%	0%	No	Yes
			attendings (2)								
			[emergency]								
Eisenburger	2000	40	Residents (10)	56	70%	15%	70	60%	10%	No	Yes
			Fellows (10)								
			[critical care]								
Schaumann	2005	200	residents (40)	119	84%	0%	99	88%	0%	No	Yes
			[emergency]								
Schober	2008	63	medical (63) students	78	94%	16%	135	71%	43%	No*	Yes
Benkhadra	2008	40	attendings (2)	N/A	N/A	N/A	71	95%	20%	No*	Yes
			[anesthesia]								
Gulsen	2010	11	attendings (3)	88	100%	N/A	N/A	N/A	N/A	No	No
			[surgery]								
Helm	2012	30	residents (30)	95	100%	0%	N/A	N/A	N/A	No	Yes
			[anesthesia]								
Heymans	2016	60	medical (20) students	94	95%	10%	149	50%	10%	No*	No
Zagona-Prizio (this report)	2022	48	fellows (11) attendings (13)	35.5	96%^a^	20%	91.3	100%	56%	Yes	No
			[critical care]								

# designates number of cadavers; PTt, puncture-to-tube time; N/A, not applicable or not available; only English language reports are listed

* endoscopy was performed and/or analyzed after training procedures to verify success and/or to assess for wounds

^a^ Success rate defined as placement of endotracheal tube within tracheal lumen

Analysis of the 24 total bronchoscopy recordings for complications revealed eight instances of sharp instruments (trocar needles and scalpels) puncturing the posterior tracheal wall (38% overall injury rate summing both techniques; example Fig 3A–3E). Of these, 3 were from scalpels (20% of scalpel technique recording) and 5 were from needles (56% of Seldinger recordings). Sharp instruments reached potentially dangerous insertion depths beyond the midpoint of the anterior-posterior diameter of the trachea in 58.3% of videos. During the session, two other unanticipated complications were detected on the monitor when a bougie inserted adjacent to the trachea (**Fig 3E**) and an endotracheal tube inserted at a vertical angle caught on the posterior tracheal wall and was unable to be advanced (not shown; this was the same trainee’s attempt and was the sole unsuccessful airway placement of the session). Analysis of the trio data revealed that the third member of the trio (who saw the live video feed of their two colleagues before their own attempt) injured the posterior tracheal wall significantly less often (*P = 0*.*0395*) than the preceding trio members (**Table 3**).

**Table 3:** Evidence for a bronchoscopic training effect from live viewing of colleagues’ sharp instrument errors.

	Trainee 1	Trainee 2	Trainee 3
**Hit Posterior Wall**	4	4	0
**Avoided Posterior Wall**	3	3	7

Chi^2^ p value 0.0395 (Trainee 3 vs Trainees 1–2)

Data is from 7 trios of trainees, one trio of trainees’ videos were not timestamped and therefore excluded from this analysis.

The response rate for the pre- and post-session survey was 100% (24 out of 24 participants). On average, the participants reported simulating 1.5±0.9 cricothyrotomies during residency or fellowship, with 50% of respondents only simulating 1 cricothyrotomy in prior training. Seventy-one percent of participants had never practiced a cricothyrotomy on a cadaver. The average number of cricothyrotomies performed on a real patient in a clinical situation during residency or fellowship for respondents was 0.3±0.8, with 79% of participants never performing a cricothyrotomy on a real patient (**S1 Table**).

All the of participants found the cadaveric training session quite or extremely helpful. 95.8% of participants found the simulation training video quite or extremely helpful. Bronchoscopic visualization of the bougie and tubes in the trachea and video playback to assess depth of incision were rated as quite helpful or extremely helpful by 83.3% and 91.7% of participants, respectively.

Wilcoxon signed-rank tests indicated a statistically significant average increase in confidence (64.4%, Z = -4.34, *P*<0.001) ***and*** average decrease in performance anxiety (-11.6%, Z = 2.135, *P* = 0.0328) associated with participation in the training session (**Table 4**). Univariate regression analysis revealed no statistically significant relation between self-reported confidence or anxiety with performing cricothyrotomies and experience with the procedure on real or simulated patients (**S2 Table, S1 Fig**).

**Table 4:** Quantitative pre- and post-session survey results.

	Mean Likert score				
Survey Question	Pre-session	Post-session	Percent Change	Z score	*P*-value
		Mean ± SD (n = 24)			
	Mean ± SD (n = 24)				
How **confident** are you that you could successfully perform an emergent cricothyrotomy on a patient in the ICU?^a^	1.87 ± 0.68	3.08 ± 0.58	64.4%	-4.34	<0.001
How **anxious** do you feel about the possibility of performing an emergent cricothyrotomy on a patient in the ICU?^b^	2.88 ± 0.74	2.54 ± 0.78	-11.6%	2.135	0.0328

SD = standard deviation

^a^ 4-point Likert scale for confidence: Not at all confident (1), Slightly confident (2), Moderately confident (3), Extremely confident (4).

^b^ 4-point Likert scale for anxiety: Not anxious at all (1), Slightly anxious (2), Moderately anxious (3), Extremely anxious (4).

## Discussion

Cricothyrotomies are life-saving procedures that are infrequently performed by non-surgeons [31]. Effective training and re-training is important for successful execution of this uncommon procedure [32], but the number of cricothyrotomies practiced by providers varies depending on their training program. As cricothyrotomies become rarer with advancements in fiberoptic and video intubation methods, it is likely that the number of cricothyrotomy training opportunities will continue to decrease [9–12, 15]. Therefore, it is critical to maximize the impact of each training session. In this study we demonstrated the feasibility, potential benefits, and acceptance of incorporating the innovation of bronchoscopic feedback to trainees during emergency cricothyrotomy training.

Bronchoscopic imaging provides trainees with useful data for minimizing injuries and dangerous sharp instrument technique. Several recent studies have shown utility in using video monitoring during simulation trainings to assess quality of simulation, complications, and formative skill testing [33–35]. Endoscopy has been shown to be accurate in detecting posterior tracheal wall injuries caused by kit cricothyrotomies as confirmed by cadaveric dissection [24]. Moreover, live bronchoscopy is now recommended as standard of care during percutaneous Seldinger percutaneous tracheostomy surgeries to prevent posterior tracheal wall violations and other injuries [17].

Results from our study demonstrate the added benefit of real-time identification of sharp instrument depths touching the posterior tracheal wall, as well as identifying those with only a high potential for injury (exceeding the midpoint of the tracheal anterior-posterior diameter) that don’t result in injury in a cadaver but might result in injury to alive, non-intubated patients with labored respiratory efforts. Importantly, posterior tracheal wall puncture was more common in our study with trocar needles during the Seldinger technique than previously reported (**Table 2**). We postulate this high rate reflects small needle punctures that may not have been observed in previous reports where cadavers were dissected for injury evaluations, as such small injuries may be difficult to visualize or to differentiate from tracheostomy tube abrasions that occur during placement. We propose that bronchoscopic monitoring obviates the need for posterior tracheal wall injury scoring by pathologic dissection of donors, which augments the training session by allowing participants to adjust their sharp instrument insertion technique during the session.

The procedural times reported in our study are among the lowest reported for the surgical technique, which generally is faster and has lower injury and non-completion rates than the Seldinger technique in previous reports [18–27]. These lower rates highlight the potential benefits of adding bronchoscopy to the training—as trainees were able to use it as a tool during their observations of colleagues’ attempts. However, having an attending otolaryngology surgeon instruct each trainee during the procedure also likely influenced the fast completion times we observed with the surgical technique. Furthermore, trainees were provided with multiple opportunities for bronchoscopic feedback, both on their own techniques (during the session for notification of posterior wall tracheal injury, and afterward for the entire technique) and through watching their colleagues’ videos in realtime. The benefit of the video footage is suggested by the session trio data where the third trainee (with opportunity to watch two procedures with bronchoscopic footage beforehand) hit the posterior wall significantly less often than the two preceding trainees (**Table 3**). This preliminary data is promising but was will need to be confirmed in future studies analyzing the training effects of bronchoscopy in larger groups.

Another less common cricothyrotomy complication is securing a false airway, which can lead to a significant loss of time during the procedure and result in devastating consequences for the patient [36]. In published data from other training programs, such events were most commonly detected only by later pathologic dissection of training cadavers (**Table 2**). In our study, one bougie was inserted adjacent to, but not within the trachea, which was noted on the bronchoscope monitor by the airway expert before it resulted in the completion of a false airway. The use of live endoscopic monitoring therefore allowed for detection of this clinically devastating scenario with immediate feedback to the trainee, who had otherwise been under the impression that their procedure was successful.

Time spent securing the airway during a cricothyrotomy is paramount due to impending risks of brain injury and cardiac arrest from prolonged hypoxemia. PTt for the scalpel technique in our study was among the fastest reported (**Table 2**) [18–27], which likely reflects several factors including a colleague assisting with passing the bougie and endotracheal tube (replicating clinical scenarios in a hospital) and airway expert coaching. PTt for the scalpel method in this study was also significantly faster than the Seldinger method, which may reflect the simplicity of the scalpel technique and that it was assisted. In contrast to the complication rate being lower for the trainee in a trio in our study, PTt was not different for the third trainee in a trio. This suggests that highlighting complications from sharp instrument depth to trainees may be the greatest benefit for the bronchoscopic component of our program and that it may have no training effect on procedure duration.

Finally, this training was feasible in that it was cost effective, required few operators to complete setup in a reasonable time (<15 min/donor), and met or exceeded prior reports for procedural duration and completion rates (**Table 2**). It also met our pre-determined quality measures through achieving the first two levels of the Kirkpatrick model for evaluating a training program as it was helpful to all participants and resulted in significantly increased confidence and decreased anxiety [28]. Future longitudinal studies will be needed to investigate whether our program meets levels three and four of the model, with participants applying their trained skills in the field with improved outcomes.

This pilot study had limitations. The program was initiated during the COVID-19 pandemic which led to campus contact rules that altered our initial plan for larger training groups down to a size of three per group, which limited the opportunities for observing live bronchoscopic feedback and learning from colleagues’ procedures. We also did not have a parallel control group of non-bronchoscopy trainees as our primary outcome was to demonstrate feasibility, acceptability, and quality of adding bronchoscopy to an emergent cricothyrotomy training program. Moreover, as this was the first-ever emergent cricothyrotomy training program for non-surgeons on our campus, we did not have a historical control group to use for comparison during our data analysis, thus we can only present our results in the context of other programs that have published their data. Importantly, cost and availability of cadaveric donors will always limit the goal for every trainee to have an intact cricothyroid membrane (not previously punctured) to practice each technique. Future studies comparing outcomes with a parallel non-bronchoscopy group will be needed to clarify the educational benefit of bronchoscopy in optimizing cricothyrotomy training. We also did not formally assess for differences in technique after trainees viewed their procedural videos as subsequent procedures (focusing on any unsafe or nonefficient steps) were performed through the same incision due to cadavers being in limited supply. Finally, we did not perform pathologic examinations to confirm injuries as our donors were soon thereafter used for graduate student neck dissections. Thus, we could not estimate potentially missed wounds previously reported in other cricothyrotomy training programs such as cricoid or laryngeal cartilage rupture or large vessel injury. Moreover, all cadaveric training programs are limited by not being able to replicate the bleeding, chaotic environment, coughing on airway entry, and patient movement that would affect success rates and complications in real patients.

Now that we have demonstrated feasibility, quality, utility in identifying injuries, and early evidence of a training effect from viewing colleagues’ live bronchoscopic images, future studies will be needed to confirm the benefits of adding bronchoscopic feedback to training. Such studies would include bronchoscopic recordings for all trainees, with randomization to receiving endoscopic feedback or not (on their own attempts, and on colleague attempts), with sufficient statistical power to assess for differences in these outcomes and in post-training confidence between groups.

## Conclusion

Cadaveric emergency cricothyrotomy training supplemented with the novel element of real-time bronchoscopy is a feasible and well-accepted innovation that meets efficient time standards for procedures reported by previous studies. Furthermore, it allows for detecting complications during the session such as potentially dangerous insertion depths that might otherwise be missed with feedback from post-procedure dissections alone. Bronchoscopic feedback allows trainees the opportunity to refine their techniques during sessions and practice safer sharp instrument depths. Additionally, trainees showed improved confidence and decreased anxiety in performing the procedures suggesting that quality is maintained when adding in this training modality. Bronchoscopic-enhancement of cricothyrotomy programs is an effective tool for maximizing each training session and may result in fewer complications when trainees are called upon to perform these emergency procedures in future clinical settings. A bronchoscopically-enhanced curriculum that follows our model can be feasibly employed at other institutions and organizations to optimize training of this uncommonly performed, but life-saving procedure.

## Supporting information

S1 Table Demographic characteristics of trainees in the three sessions. (DOCX)

S2 Table Univariate regression analysis for pre- and post-session changes in confidence and anxiety among participants associated with their experience level in performing a cricothyrotomy on a real or simulated patient. (DOCX)

S1 Fig Pre- and post-training confidence and anxiety levels based on participants’ level of training. No significant difference observed for pre- and post-training confidence or anxiety between attending vs fellow level of training. ** Significant average increase in confidence (64.4%, P<0.001) across all participants. * Significant average decrease in anxiety (-11.6%, P = 0.0328) across all participants.
(TIF)

## References

[1] Heidegger T. Management of the Difficult Airway. N Engl J Med. 2021;384(19):1836–47. Epub 2021/05/13. doi: 10.1056/NEJMra1916801 . 33979490

[2] Apfelbaum JL, Hagberg CA, Caplan RA, Blitt CD, Connis RT, Nickinovich DG, et al. Practice guidelines for management of the difficult airway: an updated report by the American Society of Anesthesiologists Task Force on Management of the Difficult Airway. Anesthesiology. 2013;118(2):251–70. Epub 2013/02/01. doi: 10.1097/ALN.0b013e31827773b2 . 23364566

[3] Bair AE, Filbin MR, Kulkarni RG, Walls RM. The failed intubation attempt in the emergency department: analysis of prevalence, rescue techniques, and personnel. J Emerg Med. 2002;23(2):131–40. Epub 2002/10/03. doi: 10.1016/s0736-4679(02)00501-2 . 12359280

[4] DeVore EK, Redmann A, Howell R, Khosla S. Best practices for emergency surgical airway: A systematic review. Laryngoscope Investig Otolaryngol. 2019;4(6):602–8. Epub 2020/01/01. doi: 10.1002/lio2.314 ; PubMed Central PMCID: PMC6929583. 31890877 PMC6929583

[5] Macedo MB, Guimaraes RB, Ribeiro SM, Sousa KM. Emergency cricothyrotomy: temporary measure or definitive airway? A systematic review. Rev Col Bras Cir. 2016;43(6):493–9. Epub 2017/03/09. doi: 10.1590/0100-69912016006010 . 28273224

[6] Hubble MW, Wilfong DA, Brown LH, Hertelendy A, Benner RW. A meta-analysis of prehospital airway control techniques part II: alternative airway devices and cricothyrotomy success rates. Prehosp Emerg Care. 2010;14(4):515–30. Epub 2010/09/03. doi: 10.3109/10903127.2010.497903 . 20809690

[7] Moroco AE, Armen SB, Goldenberg D. Emergency Cricothyrotomy: A 10-Year Single Institution Experience. Am Surg. 2021:3134821995075. Epub 2021/02/11. doi: 10.1177/0003134821995075 . 33566678

[8] Katayama A, Nakazawa H, Tokumine J, Lefor AK, Watanabe K, Asao T, et al. A high-fidelity simulator for needle cricothyroidotomy training is not associated with increased proficiency compared with conventional simulators: A randomized controlled study. Medicine (Baltimore). 2019;98(8):e14665. Epub 2019/03/01. doi: 10.1097/MD.0000000000014665 ; PubMed Central PMCID: PMC6408010. 30813212 PMC6408010

[9] Reeder TJ, Brown CK, Norris DL. Managing the difficult airway: a survey of residency directors and a call for change. J Emerg Med. 2005;28(4):473–8. Epub 2005/04/20. doi: 10.1016/j.jemermed.2004.11.027 . 15837035

[10] Makowski AL. A survey of graduating emergency medicine residents’ experience with cricothyrotomy. West J Emerg Med. 2013;14(6):654–61. Epub 2014/01/02. doi: 10.5811/westjem.2013.7.18183 ; PubMed Central PMCID: PMC3876318. 24381695 PMC3876318

[11] Iserson KV. Postmortem procedures in the emergency department: using the recently dead to practise and teach. J Med Ethics. 1993;19(2):92–8. Epub 1993/06/01. doi: 10.1136/jme.19.2.92 ; PubMed Central PMCID: PMC1376195. 8331644 PMC1376195

[12] Takayesu JK, Peak D, Stearns D. Cadaver-based training is superior to simulation training for cricothyrotomy and tube thoracostomy. Intern Emerg Med. 2017;12(1):99–102. Epub 2016/03/30. doi: 10.1007/s11739-016-1439-1 . 27021389

[13] Mandell D, Orebaugh SL. A Porcine Model for Learning Ultrasound Anatomy of the Larynx and Ultrasound-Guided Cricothyrotomy. Simul Healthc. 2019;14(5):343–7. Epub 2019/04/11. doi: 10.1097/SIH.0000000000000364 . 30969269

[14] Wilson AB, Barger JB, Perez P, Brooks WS. Is the supply of continuing education in the anatomical sciences keeping up with the demand? Results of a national survey. Anat Sci Educ. 2018;11(3):225–35. Epub 2017/09/15. doi: 10.1002/ase.1726 . 28906598

[15] Legoux C, Gerein R, Boutis K, Barrowman N, Plint A. Retention of Critical Procedural Skills After Simulation Training: A Systematic Review. AEM Educ Train. 2021;5(3):e10536. Epub 2021/06/09. doi: 10.1002/aet2.10536 ; PubMed Central PMCID: PMC8166305. 34099989 PMC8166305

[16] Simon M, Metschke M, Braune SA, Puschel K, Kluge S. Death after percutaneous dilatational tracheostomy: a systematic review and analysis of risk factors. Crit Care. 2013;17(5):R258. Epub 2013/10/31. doi: 10.1186/cc13085 ; PubMed Central PMCID: PMC4056379. 24168826 PMC4056379

[17] Hashimoto DA, Axtell AL, Auchincloss HG. Percutaneous Tracheostomy. N Engl J Med. 2020;383(20):e112. Epub 2020/10/29. doi: 10.1056/NEJMvcm2014884 . 33113296

[18] Johnson DR, Dunlap A, McFeeley P, Gaffney J, Busick B. Cricothyrotomy performed by prehospital personnel: a comparison of two techniques in a human cadaver model. Am J Emerg Med. 1993;11(3):207–9. Epub 1993/05/01. doi: 10.1016/0735-6757(93)90125-u . 8489658

[19] Holmes JF, Panacek EA, Sakles JC, Brofeldt BT. Comparison of 2 cricothyrotomy techniques: standard method versus rapid 4-step technique. Ann Emerg Med. 1998;32(4):442–6. Epub 1998/10/17. doi: 10.1016/s0196-0644(98)70173-8 . 9774928

[20] Chan TC, Vilke GM, Bramwell KJ, Davis DP, Hamilton RS, Rosen P. Comparison of wire-guided cricothyrotomy versus standard surgical cricothyrotomy technique. J Emerg Med. 1999;17(6):957–62. Epub 1999/12/14. doi: 10.1016/s0736-4679(99)00123-7 . 10595879

[21] Eisenburger P, Laczika K, List M, Wilfing A, Losert H, Hofbauer R, et al. Comparison of conventional surgical versus Seldinger technique emergency cricothyrotomy performed by inexperienced clinicians. Anesthesiology. 2000;92(3):687–90. Epub 2000/03/17. doi: 10.1097/00000542-200003000-00012 . 10719947

[22] Schaumann N, Lorenz V, Schellongowski P, Staudinger T, Locker GJ, Burgmann H, et al. Evaluation of Seldinger technique emergency cricothyroidotomy versus standard surgical cricothyroidotomy in 200 cadavers. Anesthesiology. 2005;102(1):7–11. Epub 2004/12/25. doi: 10.1097/00000542-200501000-00005 . 15618780

[23] Schober P, Hegemann MC, Schwarte LA, Loer SA, Noetges P. Emergency cricothyrotomy-a comparative study of different techniques in human cadavers. Resuscitation. 2009;80(2):204–9. Epub 2008/12/09. doi: 10.1016/j.resuscitation.2008.10.023 . 19058897

[24] Benkhadra M, Lenfant F, Nemetz W, Anderhuber F, Feigl G, Fasel J. A comparison of two emergency cricothyroidotomy kits in human cadavers. Anesth Analg. 2008;106(1):182–5, table of contents. Epub 2008/01/01. doi: 10.1213/01.ane.0000296457.55791.34 . 18165576

[25] Gulsen S, Unal M, Dinc AH, Altinors N. Clinically correlated anatomical basis of cricothyrotomy and tracheostomy. J Korean Neurosurg Soc. 2010;47(3):174–9. Epub 2010/04/10. doi: 10.3340/jkns.2010.47.3.174 ; PubMed Central PMCID: PMC2851084. 20379468 PMC2851084

[26] Helm M, Hossfeld B, Jost C, Lampl L, Bockers T. Emergency cricothyroidotomy performed by inexperienced clinicians—surgical technique versus indicator-guided puncture technique. Emerg Med J. 2013;30(8):646–9. Epub 2012/07/31. doi: 10.1136/emermed-2012-201493 ; PubMed Central PMCID: PMC3717590. 22843552 PMC3717590

[27] Heymans F, Feigl G, Graber S, Courvoisier DS, Weber KM, Dulguerov P. Emergency Cricothyrotomy Performed by Surgical Airway-naive Medical Personnel: A Randomized Crossover Study in Cadavers Comparing Three Commonly Used Techniques. Anesthesiology. 2016;125(2):295–303. Epub 2016/06/09. doi: 10.1097/ALN.0000000000001196 . 27275669

[28] Kirkpatrick DL. Evaluating training programs: the four levels. 2nd ed. San Francisco, Calif.: Berrett-Koehler Publishers; 1998. xvii, 289 p. p.

[29] Zagona-Prizio C, Mann SE, Mayer KA, Pascoe MA, Maloney JP, Parsons B. Emergent Cricothyrotomy Training for Non-Surgeons (1.0). Zenodo2020. p. 10.5281/zenodo.4029816. PMC10035915 36952528

[30] CU Anschutz Cricothyrotomy Training Group. Emergency Cricothyrotomy Procedures: Quick Tutorial. YouTube2020. p. https://www.youtube.com/watch?v=hGI8MJNWJoc.

[31] Sagarin MJ, Barton ED, Chng YM, Walls RM, National Emergency Airway Registry I. Airway management by US and Canadian emergency medicine residents: a multicenter analysis of more than 6,000 endotracheal intubation attempts. Ann Emerg Med. 2005;46(4):328–36. Epub 2005/09/29. doi: 10.1016/j.annemergmed.2005.01.009 . 16187466

[32] Wong DT, Prabhu AJ, Coloma M, Imasogie N, Chung FF. What is the minimum training required for successful cricothyroidotomy?: a study in mannequins. Anesthesiology. 2003;98(2):349–53. Epub 2003/01/29. doi: 10.1097/00000542-200302000-00013 . 12552192

[33] Ernst A, Silvestri GA, Johnstone D, American College of Chest P. Interventional pulmonary procedures: Guidelines from the American College of Chest Physicians. Chest. 2003;123(5):1693–717. Epub 2003/05/13. doi: 10.1378/chest.123.5.1693 . 12740291

[34] Ericsson KA. Necessity is the mother of invention: video recording firsthand perspectives of critical medical procedures to make simulated training more effective. Acad Med. 2014;89(1):17–20. Epub 2013/11/28. doi: 10.1097/ACM.0000000000000049 . 24280862

[35] Shah RT, Makaryus MR, Kumar R, Singas E, Mayo PH. Simulation Training for Critical Care Airway Management: Assessing Translation to Clinical Practice Using a Small Video-Recording Device. Chest. 2020;158(1):272–8. Epub 2020/03/03. doi: 10.1016/j.chest.2020.01.047 . 32113922

[36] Cook TM, Woodall N, Harper J, Benger J, Fourth National Audit P. Major complications of airway management in the UK: results of the Fourth National Audit Project of the Royal College of Anaesthetists and the Difficult Airway Society. Part 2: intensive care and emergency departments. Br J Anaesth. 2011;106(5):632–42. Epub 2011/03/31. doi: 10.1093/bja/aer059 . 21447489

