# Supplementary material for: Cadaveric emergency cricothyrotomy training for non-surgeons using a bronchoscopy-enhanced curriculum
Source: PLoS One. 2023 Mar 23;18(3):e0282403. doi: 10.1371/journal.pone.0282403 (PMC10035915; doi:10.1371/journal.pone.0282403)
Supplement: S1 Table — (DOCX) [file pone.0282403.s001.docx]

| **S1 Table. Demographic characteristics of trainees in the three sessions** | | | | | |
| --- | --- | --- | --- | --- | --- |
| **Trainee number** | **Year of fellowship** | **Years post-fellowship** | **Simulated* CTs during fellowship** | **Real patient CTs during fellowship** | **Cadaveric CTs before this training** |
| 1 | N/A | 3 | 3 | 2 | 2 |
| 2 | N/A | 2 | 3 | 0 | 1 |
| 3 | N/A | 13 | 1 | 3 | 1 |
| 4 | N/A | 3 | 1 | 0 | 0 |
| 5 | N/A | 12 | 2 | 0 | 0 |
| 6 | N/A | 8 | 1 | 0 | 0 |
| 7 | 3 | N/A | 1 | 0 | 0 |
| 8 | 4 | N/A | 3 | 0 | 0 |
| 9 | 3 | N/A | 2 | 0 | 0 |
| 10 | 4 | N/A | 2 | 0 | 0 |
| 11 | 1 | N/A | 1 | 0 | 0 |
| 12 | 2 | N/A | 3 | 0 | 0 |
| 13 | 1 | N/A | 2 | 1 | 1 |
| 14 | 3 | N/A | 1 | 1 | 0 |
| 15 | N/A | 9 | 1 | 0 | 3 |
| 16 | N/A | 11 | 0 | 0 | 2 |
| 17 | 2 | N/A | 1 | 0 | 0 |
| 18 | N/A | 12 | 1 | 1 | 0 |
| 19 | N/A | 14 | 1 | 0 | 1 |
| 20 | N/A | 8 | 2 | 0 | 0 |
| 21 | N/A | 11 | 2 | 0 | 0 |
| 22 | N/A | 10 | 0 | 0 | 0 |
| 23 | 3 | N/A | 1 | 0 | 0 |
| 24 | 2 | N/A | 1 | 0 | 0 |

Abbreviations: CT, cricothyrotomy; N/A, not applicable

* non-cadaveric cricothyrotomy simulations, such as pig tracheas or plastic models
