# Supplementary material for: Cadaveric emergency cricothyrotomy training for non-surgeons using a bronchoscopy-enhanced curriculum
Source: PLoS One. 2023 Mar 23;18(3):e0282403. doi: 10.1371/journal.pone.0282403 (PMC10035915; doi:10.1371/journal.pone.0282403)
Supplement: S2 Table — (DOCX) [file pone.0282403.s002.docx]

**S2 Table. Univariate Regression Analysis for pre- and post-Session Changes in Confidence and Anxiety among Participants associated with their Experience Level in performing a Cricothyrotomy on a real or simulated Patient.**

| **Cricothyrotomy Experience** | **Confidence** | | **Anxiety** | |
| --- | --- | --- | --- | --- |
|  | **Pre-session**  β coefficient  (95% CI) | **Post-session**  β coefficient  (95% CI) | **Pre-session**  β coefficient  (95% CI) | **Post-session**  β coefficient  (95% CI) |
| **Real patient**  (trainee N=5) | 0.082 (-0.320 to 0.483)  *P*=0.677 | 0.167 (-0.168 to 0.503)  *P*=0.311 | -0.308 (-0.725 to 0.110)  *P*=0.141 | -0.299 (-0.701 to 0.103)  *P*=0.137 |
| **Simulated patient**  (trainee N=22) | -0.088 (-0.434 to 0.258)  *P*=0.603 | 0.102 (-0.187 to 0.390)  *P*=0.471 | 0.100 (-0.259 to 0.560)  *P*=0.567 | -0.344 (-0.691 to 0.002)  *P*=0.051 |

CI= confidence interval
