# Supplementary figures and images for: Cadaveric emergency cricothyrotomy training for non-surgeons using a bronchoscopy-enhanced curriculum
Source: PLoS One. 2023 Mar 23;18(3):e0282403. doi: 10.1371/journal.pone.0282403 (PMC10035915; doi:10.1371/journal.pone.0282403)

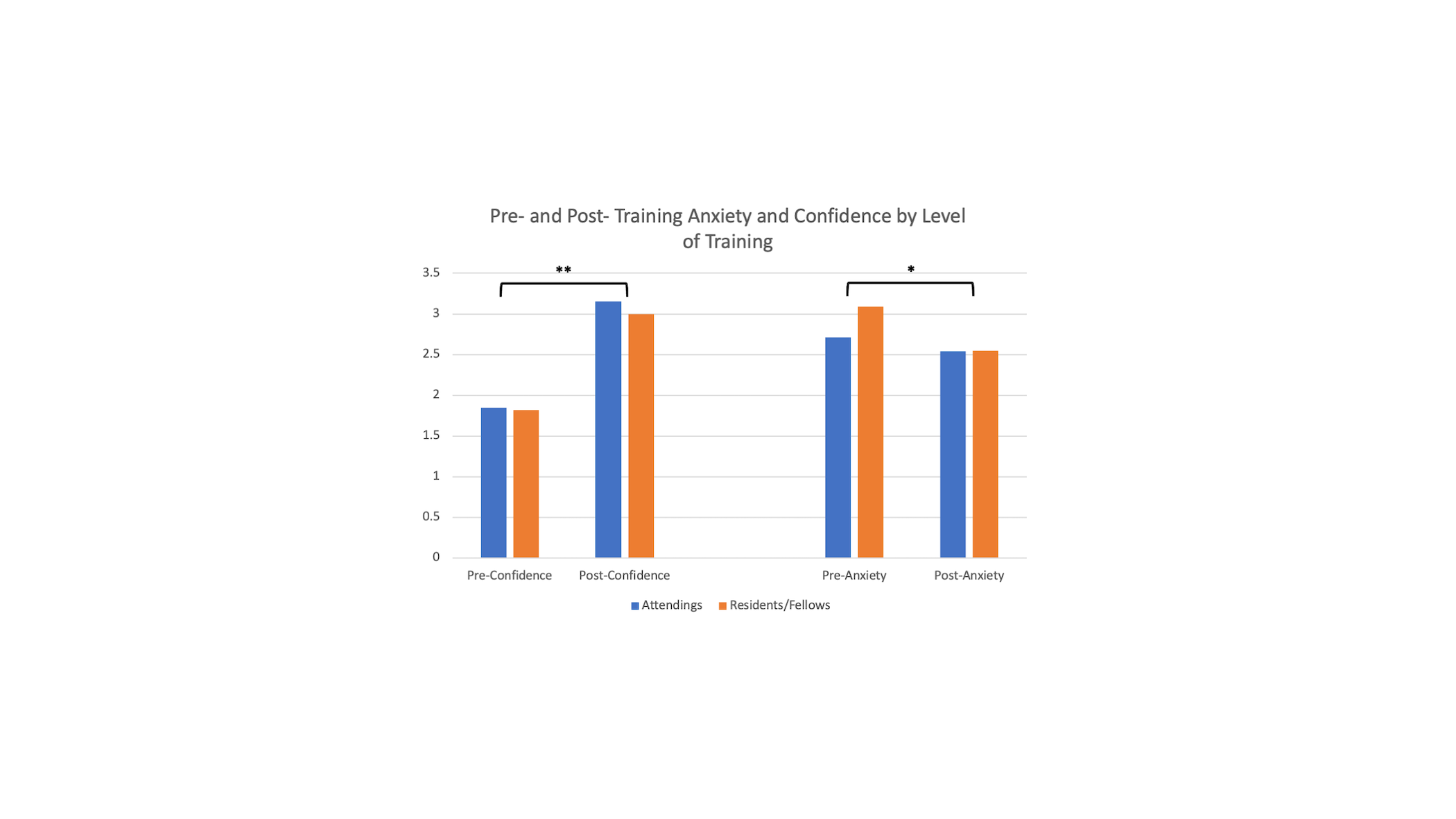

Supplement: S1 Fig — No significant difference observed for pre- and post-training confidence or anxiety between attending vs fellow level of training. ** Significant average increase in confidence (64.4%, P<0.001) across all participants. * Significant average decrease in anxiety (-11.6%, P = 0.0328) across all participants. (TIF) [file pone.0282403.s003.tif]
